# Supplementary material for: Thymine DNA glycosylase binds to R-loops and excises 5-formyl and 5-carboxyl cytosine from DNA/RNA hybrids
Source: J Biol Chem. 2026 Feb 26;302(4):111308. doi: 10.1016/j.jbc.2026.111308 (PMC13022674; doi:10.1016/j.jbc.2026.111308)
Supplement: Supplementary Material [file mmc1.pdf]

Supporting Information

Thymine DNA Glycosylase Binds to R-Loops and Excises 5-Formyl and 5-Carboxyl Cytosine from DNA/RNA Hybrids

Baiyu Zhu[1], Lakshmi S. Pidugu[3], Mary E. Cook[3], Xinyu Y. Nie[4], E. A. P. Tharaka Amarasekara[1], Jerome S. Menet[4], Alexander C. Drohat[3], and Jonathan T. Sczepanski[1,2,*]

[1] Department of Chemistry, Texas A&M University, College Station, Texas, 77843, USA
[2] Department of Biochemistry and Biophysics, Texas A&M University, College Station, Texas, 77843, USA
[3] Department of Biochemistry and Molecular Biology, University of Maryland School of Medicine, Baltimore, MD 21201, USA
[4] Department of Biology, Center For Biological Clock Research, Texas A&M University, College Station, TX 77843, USA

* To whom correspondence should be addressed. Email: jon.sczepanski@chem.tamu.edu

S1. Supplementary Figures

Figure S1

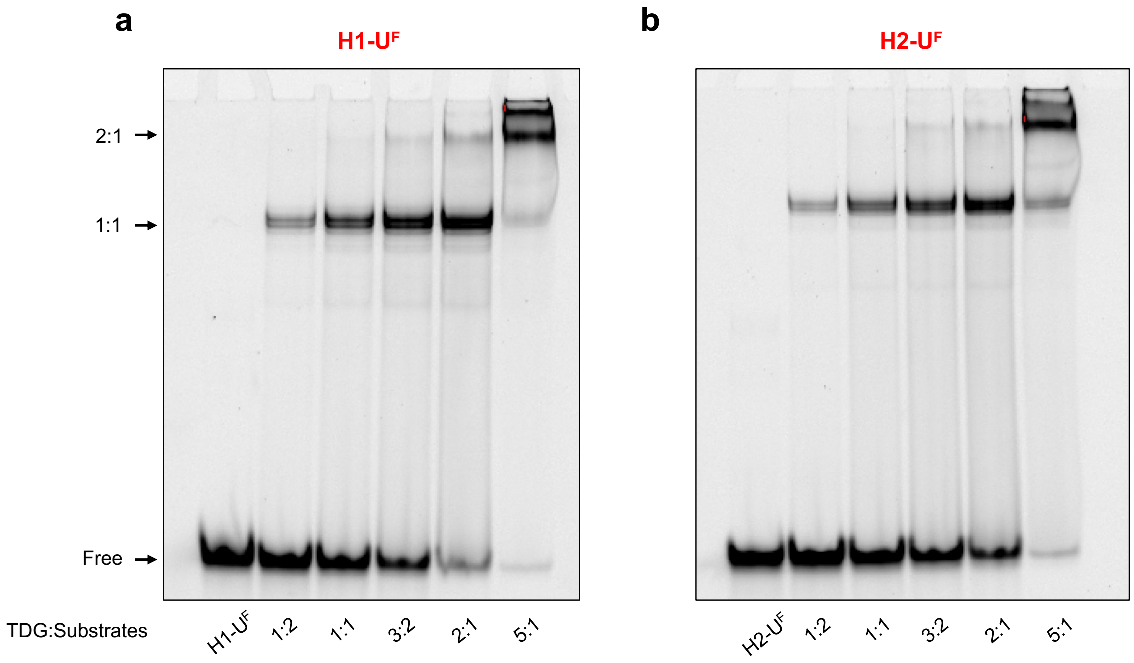

Figure S1. Representative native PAGE gels (10%, 29:1 acrylamid:bisacrylamide) showing the formation of 1:1 and 2:1 complexes of TDG (100 – 1000 nM) with either (a) H1-U^F or (b) H2-U^F. For each reaction, the indicated substrate (200 nM) was mixed with TDG in a buffer containing 100 mM NaCl, 2.5 mM $MgCl_2$, and 10 mM Tris-HCl (pH 7.5), and was incubated at 30 °C for 30 minutes. Uncropped gel images are presented in Figure S15.

Figure S2

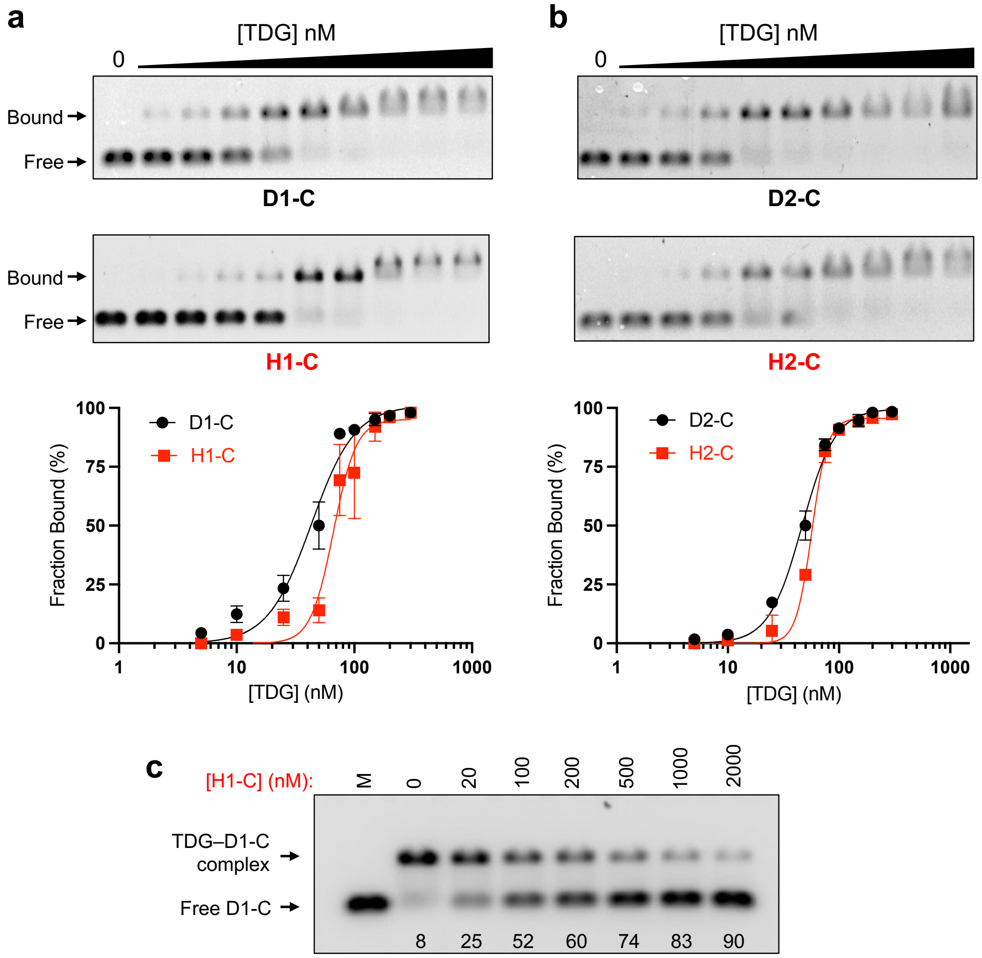

Figure S2. (a) Representative EMSA data and corresponding saturation plots for binding of TDG (0 – 300 nM) to either D1-C or H1-C (5 nM). (b) Representative EMSA data and corresponding saturation plots for binding of TDG (0 – 300 nM) to either D2-C or H2-C (5 nM). Reaction conditions are the same as those described in Figure 2b,c. Data are mean ± S.D. (n = 3). (c) DNA/RNA hybrid duplexes effectively compete with DNA/DNA duplexes for TDG binding. Preformed complexes between FAM-labeled D1-C (100 nM) and TDG (200 nM) are disrupted by unlabeled hybrid H1-C. The "M" marker indicates D1-C in the absence of TDG and hybrid. The percent free (unbound) D1-C is indicated underneath each lane. Reaction conditions are the same as those described in Figure 2b,c. Uncropped gel images are presented in Figure S16.

Figure S3

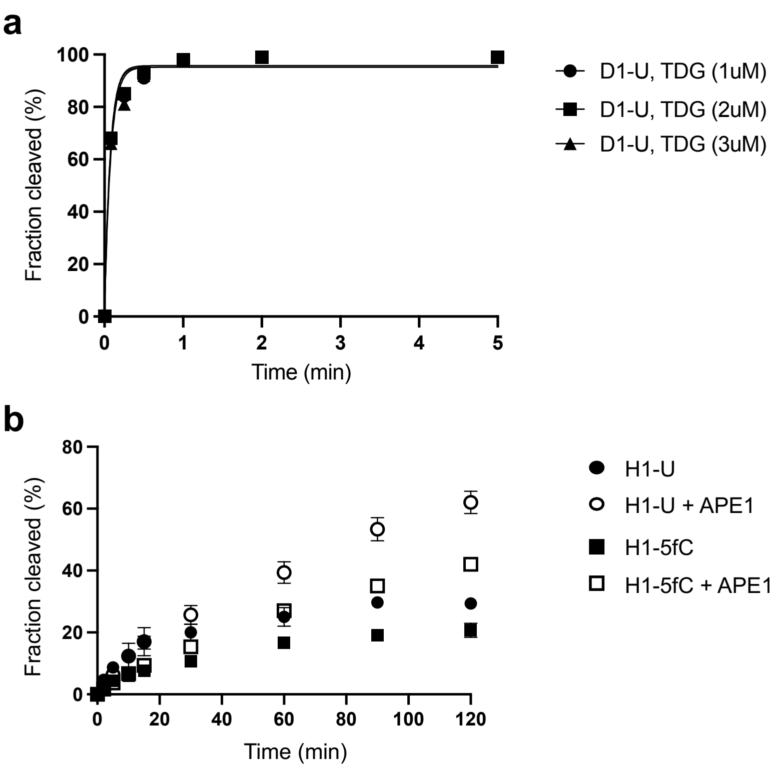

Figure S3. (a) Single-turnover kinetics using different concentrations of TDG (1000, 2000, and 3000 nM) acting on D1-U (100 nM). For each reaction, D1-U (100 nM) was mixed with TDG (1000, 2000, and 3000 nM) respectively in a buffer containing 100 mM NaCl, 10 mM Tris-HCl pH 7.5, 2.5 mM $MgCl_2$ and was incubated at 30 °C for 30 minutes. Data are mean ± S.D. (n = 3). (b) Multi-turnover kinetics of TDG (100 nM) acting on the indicated substrate (1000 nM) in the presence or absence of APE1 (100 nM). All reactions contained 100 mM NaCl, 2.5 mM $MgCl_2$, and 10 mM Tris-HCl (pH 7.5) and were carried out at 30 °C. Data are mean ± S.D. (n = 3)

Figure S4

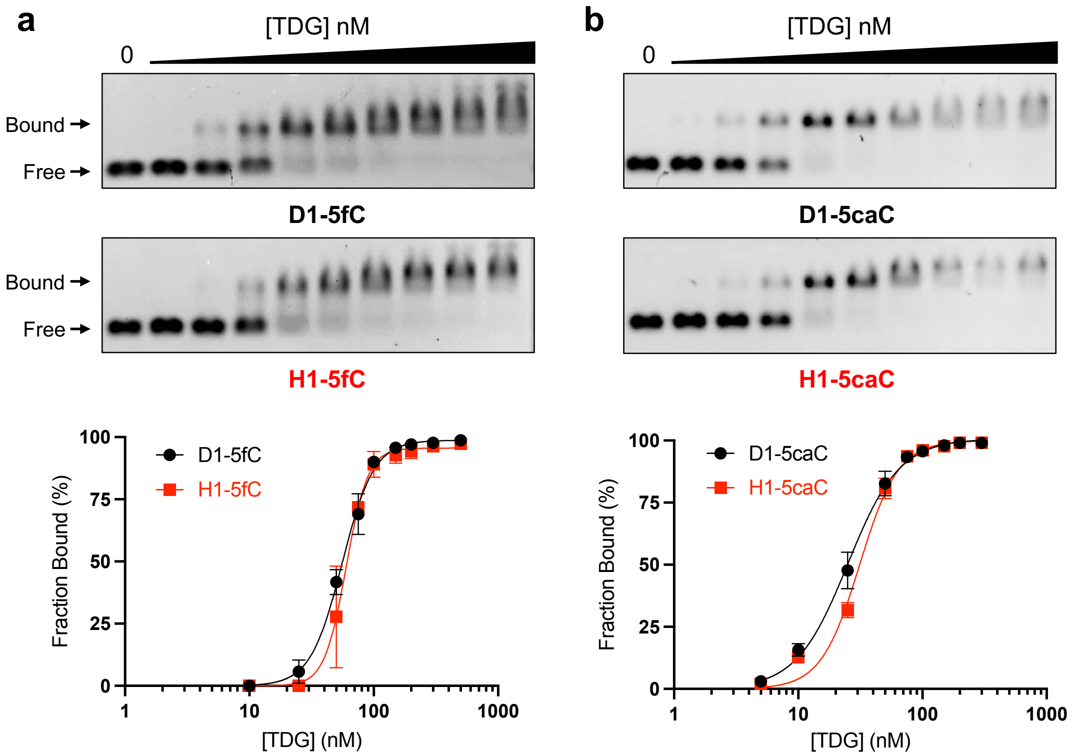

Figure S4. (a) Representative EMSA data and corresponding saturation plots for binding of TDG (0 – 300 nM) to either D1-5fC or H1-5fC (5 nM). (b) Representative EMSA data and corresponding saturation plots for binding of TDG (0 – 300 nM) to either D2-5caC or H2-5caC (5 nM). Reaction conditions are the same as those described in Figure 2b,c. Data are mean ± S.D. (n = 3). Uncropped gel images are presented in Figure S17.

Figure S5

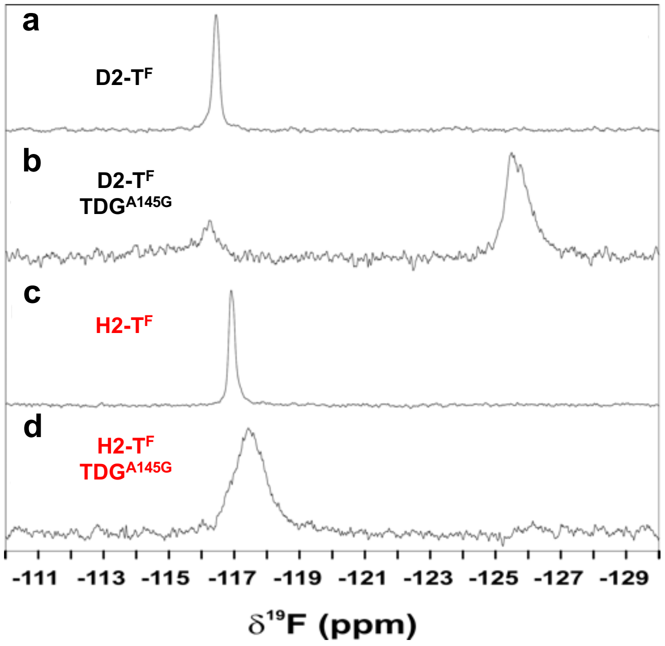

Figure S5. ^{19}F NMR spectra for D2-T^F (a,b) or H2-T^F (c,d) in the absence or presence of TDG^{A145G}. The DNA concentrations were 67 μM to 75 μM and the enzyme concentration was at least twofold greater than DNA.

Figure S6

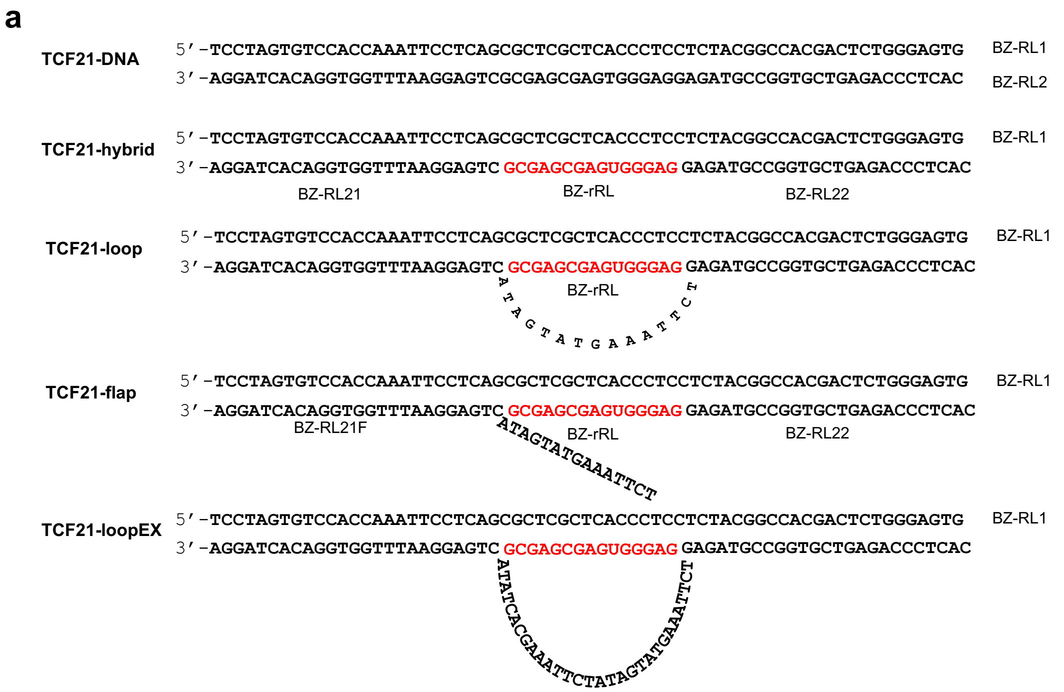

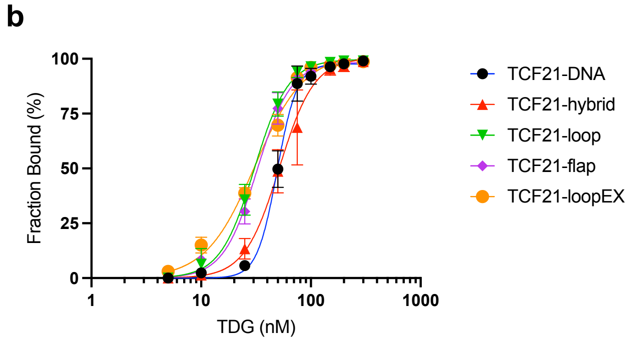

c

Substrate	K_d (nM)	95% CI	Hill Coef.
TCF21-DNA	49	47 - 51	4.8
TCF21-hybrid	52	47 - 52	2.7
TCF21-loop	31	29 - 33	2.8
TCF21-flap	33	31 - 35	2.8
TCF21-loopEX	31	29 - 33	1.9

Figure S6. (a) Schematic and sequences of all TCF21-derived substrates. Black and red colors denote DNA and RNA, respectively. (b) Saturation plots for binding of TDG (0 – 300 nM) with the indicated R-loop substrates. Reaction conditions are the same as those described in Figure 2b,c. (c) Equilibrium dissociation constats for TDG binding to the indicated substrates. 95% confidence interval (95% CI).

Figure S7

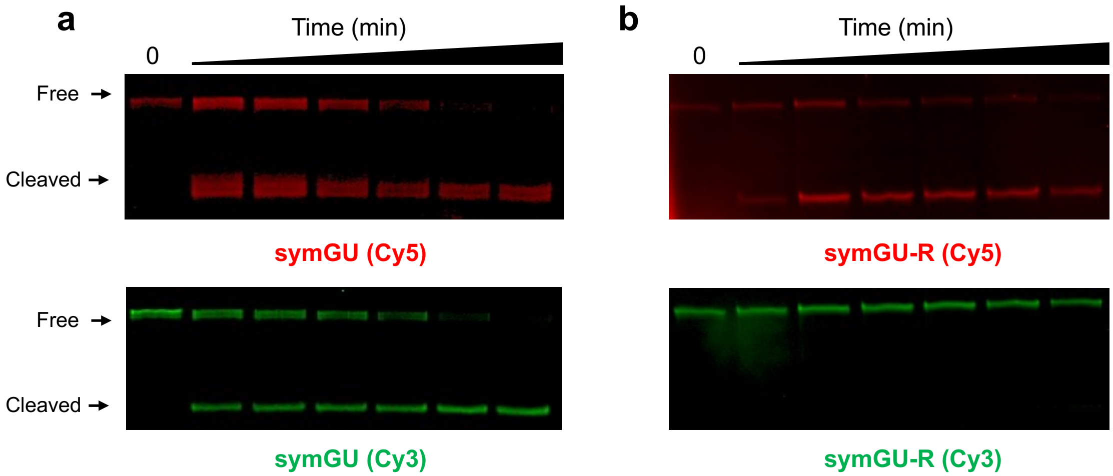

Figure S7. Single-turnover kinetics of TDG-mediated cleavage of symGU (a) or symGU-R (b) as measured by denaturing PAGE (20%, 19:1 acrylamide:bisacrylamide). Representative gels are shown, which were scanned using the indicated channel. Reaction conditions are the same as those described in Figure 3. Uncropped gel images are presented in Figure S18.

Figure S8

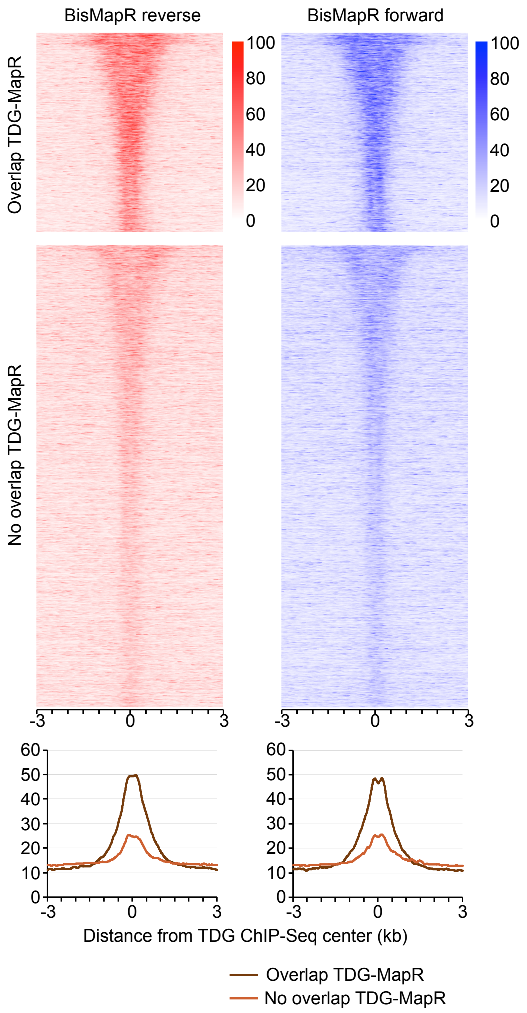

Figure S8. TDG ChIP-Seq signal is enriched at R-loops. (top) Heatmap representations of BisMapR reverse and forward signal on a window of ±3 kb around the center of TDG ChIP-seq peaks. Reads were parsed based on the overlap of TDG peaks with MapR peaks and ordered based on TDG ChIP-Seq signal, as in Figure 7a. (bottom) Quantification of BisMapR reverse and forward signal signal at TDG peaks that overlap with an R-loop (brown) or not (orange).

Figure S9

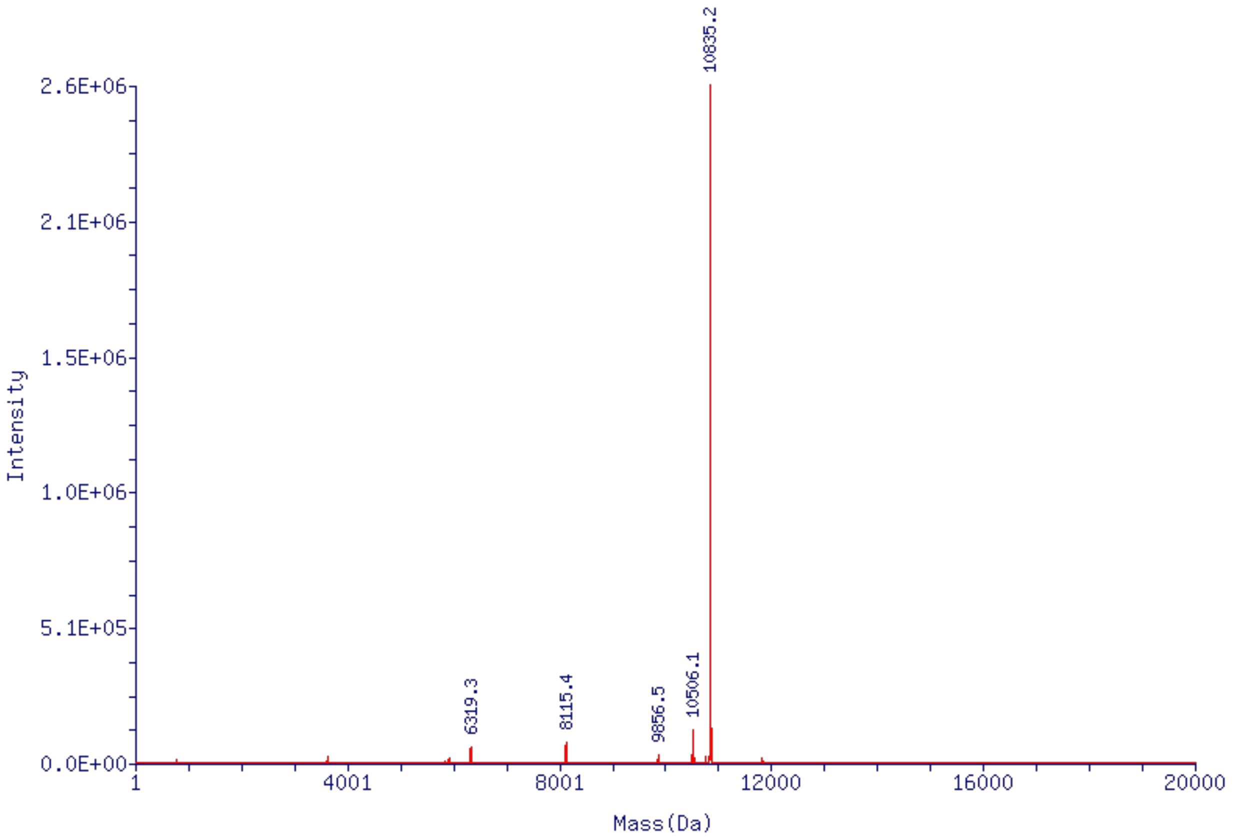

Figure S9. ESI-MS of BZ-dU^{F}FWD. Mass calculated: 10835.14 Da; Mass found: 10835.2 Da.

Figure S10

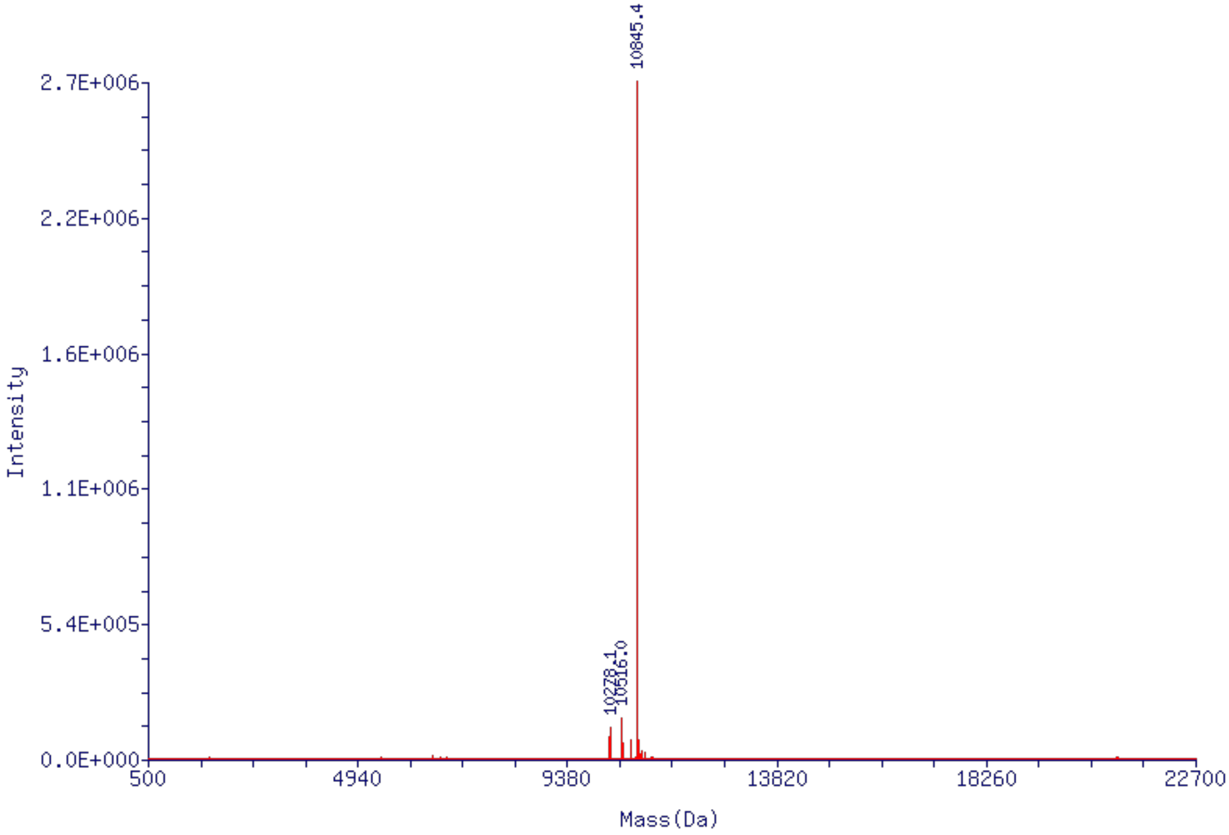

Figure S10. ESI-MS of BZ-5fCFWD. Mass calculated: 10844.17 Da, Mass found: 10845.4 Da.

Figure S11

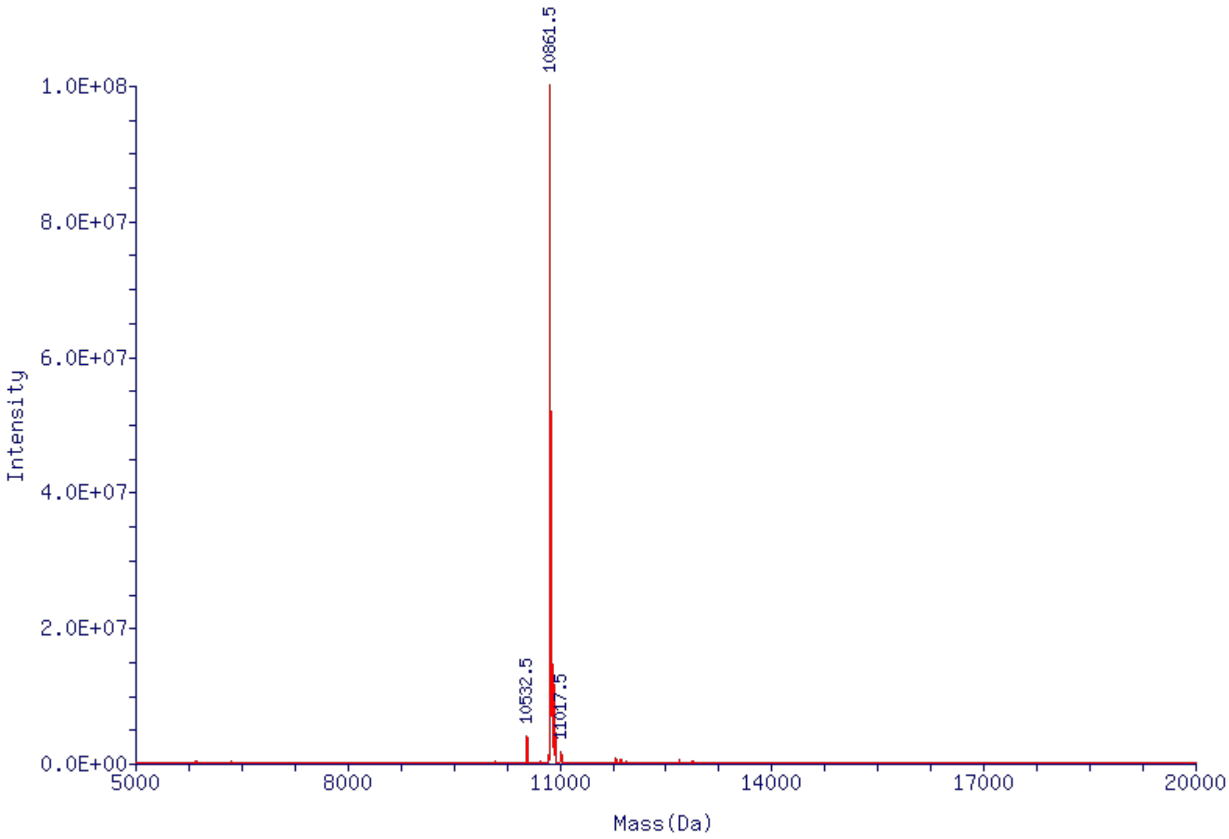

Figure S11. ESI-MS spectrum of BZ-5caCFWD. Mass calculated: 10860.17 Da, Mass found: 10861.5 Da.

Figure S12

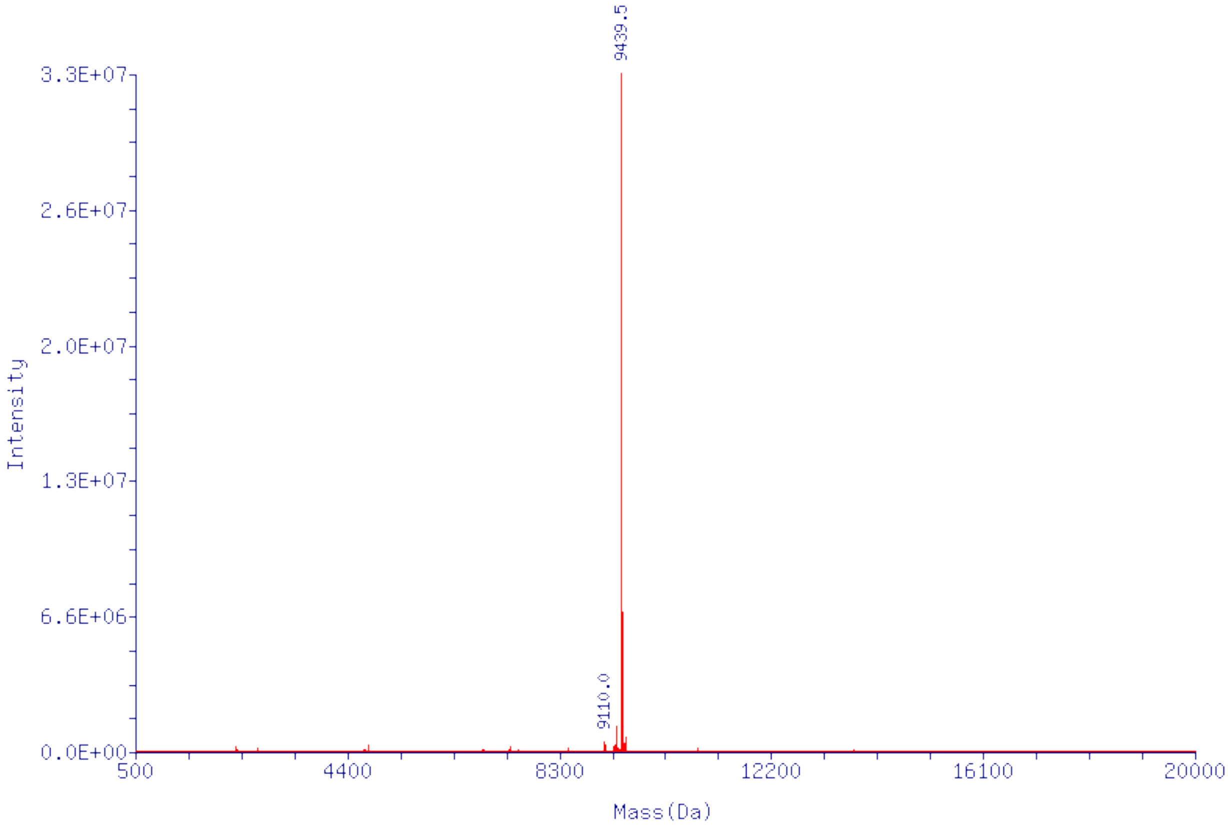

Figure S12. ESI-MS spectrum of AD-dU^{F}FWD. Mass calculated: 9439.24 Da, Mass found: 9439.5 Da.

Figure S13

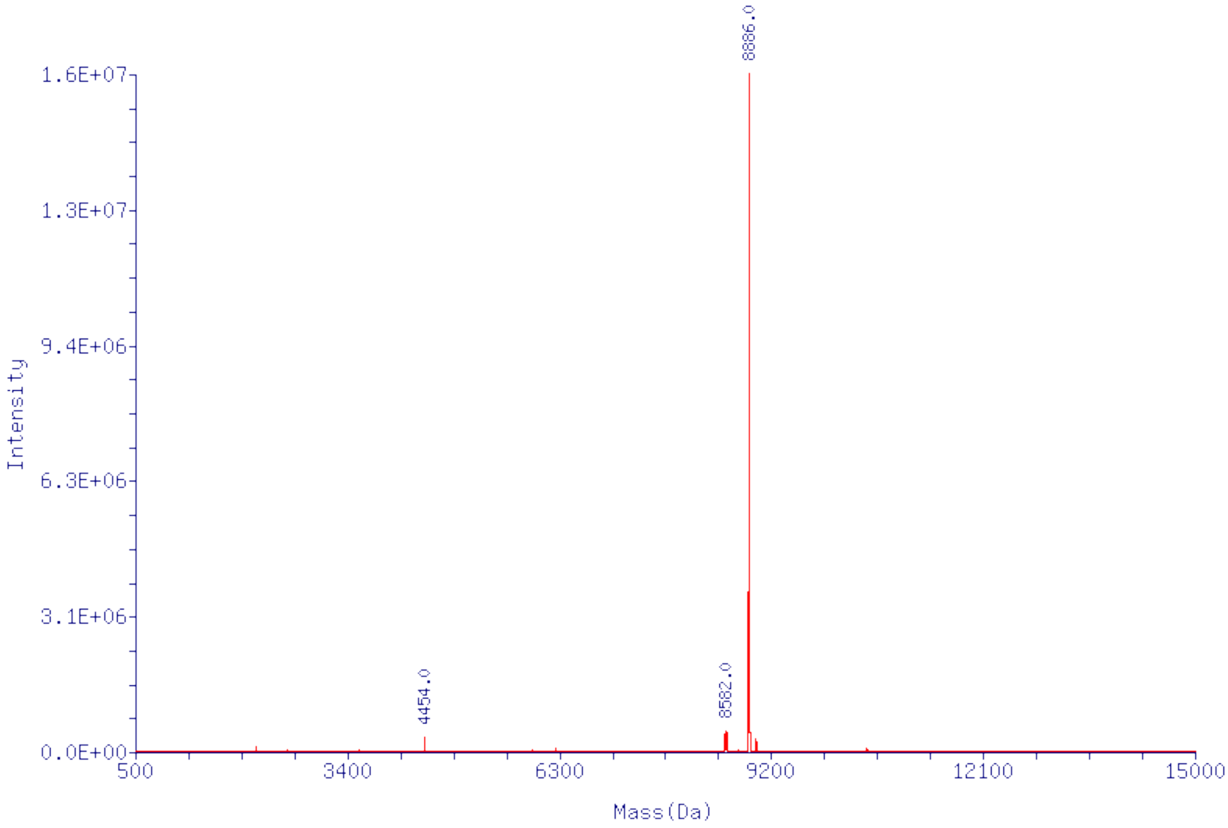

Figure S13. ESI-MS spectrum of AD-dT^{F}FWD. Mass calculated: 8886.78 Da, Mass found: 8886.0 Da.

Figure S14

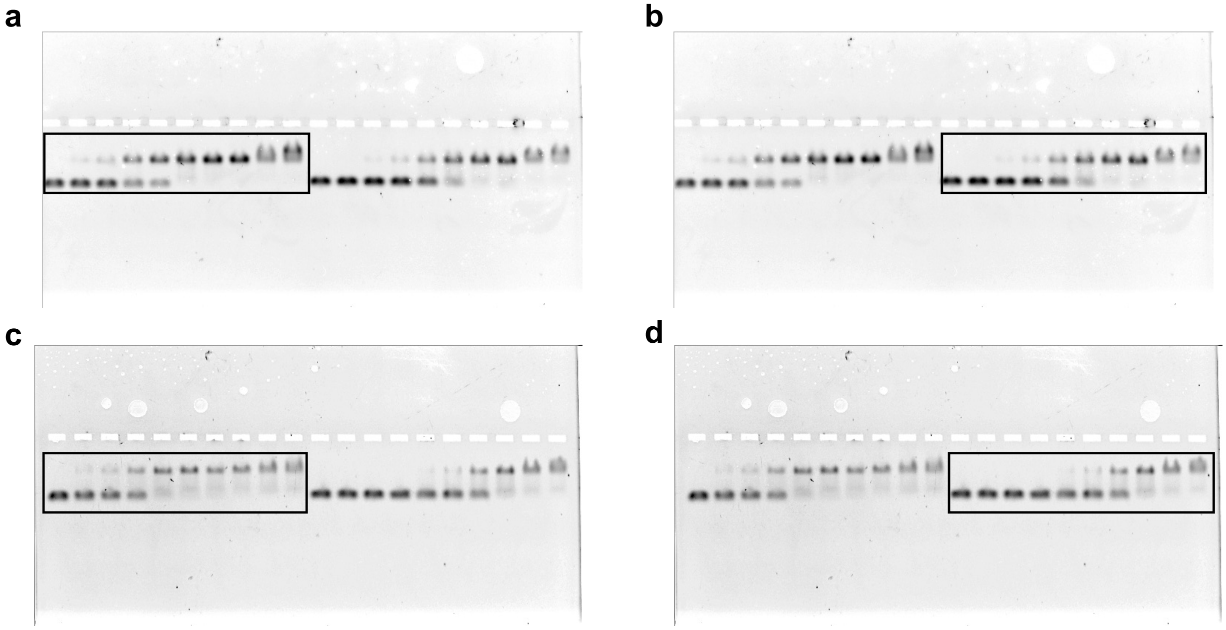

Figure S14. Uncropped gel images for the main text Figure 1b and 1c. (a) D1-U^F. (b) H1-U^F. (c) D2-U^F. (d) H2-U^F. Box regions indicate the cropped image shown in Figure 1b and 1c.

Figure S15

a

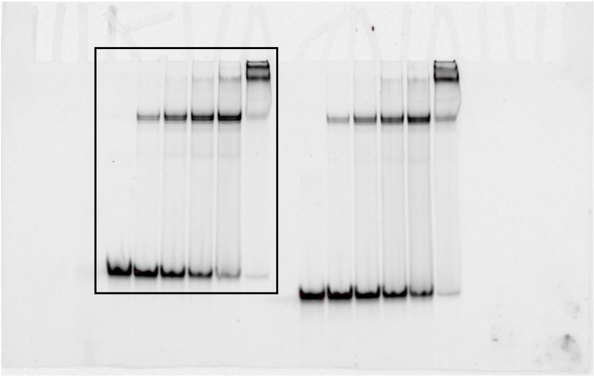

b

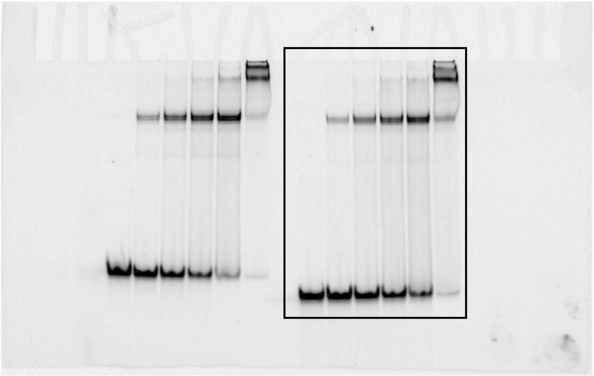

Figure S15. Uncropped gel images for Figure S1. (a) H1-U^F. (b) H2-U^F. Box regions indicate the cropped image shown in Figure S1.

Figure S16

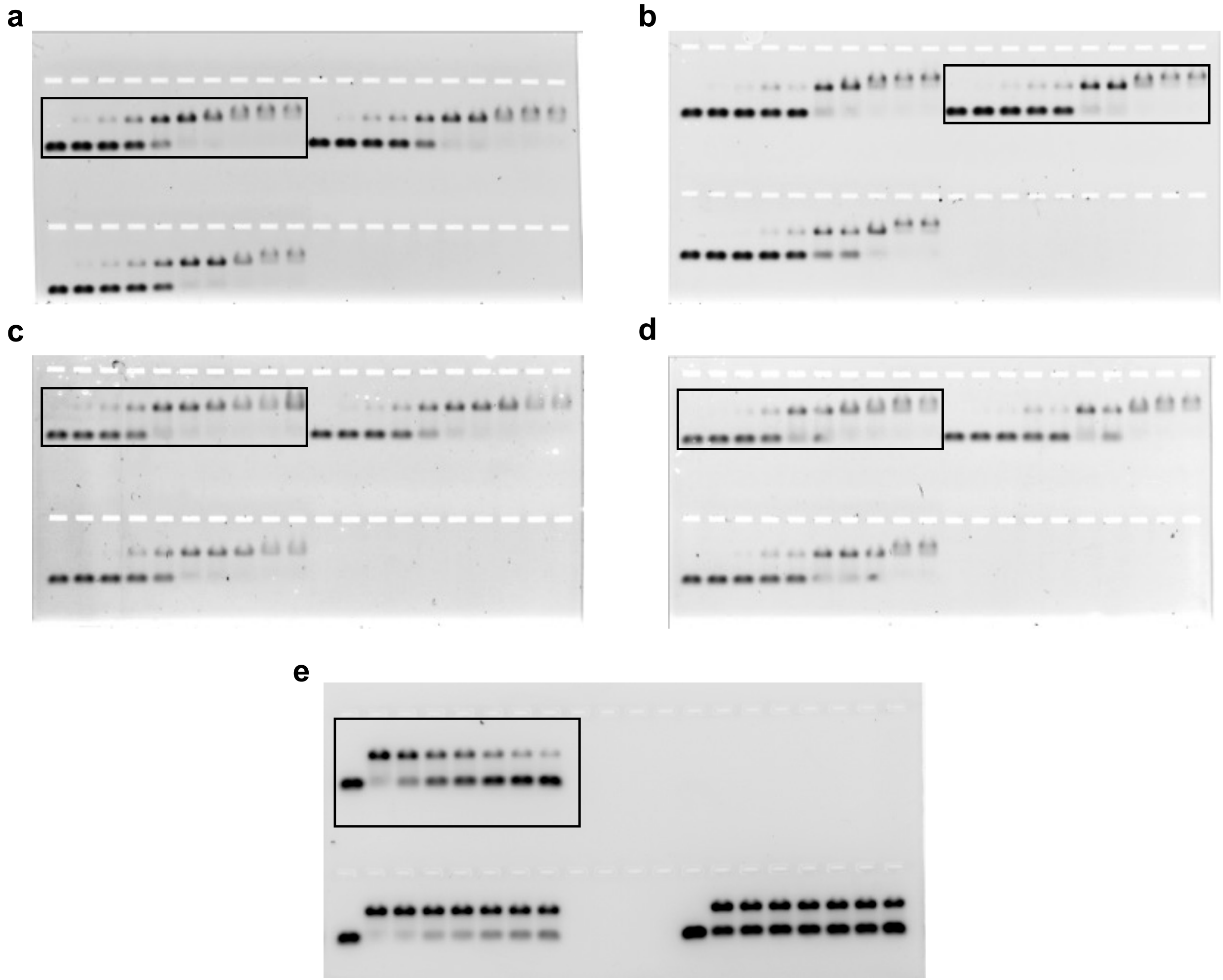

Figure S16. Uncropped gel images for Figure S2. (a) D1-C. (b) H1-C. (c) D2-C. (d) H2-C. (e) Competition experiment in panel S2c. Box regions indicate the cropped image shown in Figure S2.

Figure S17

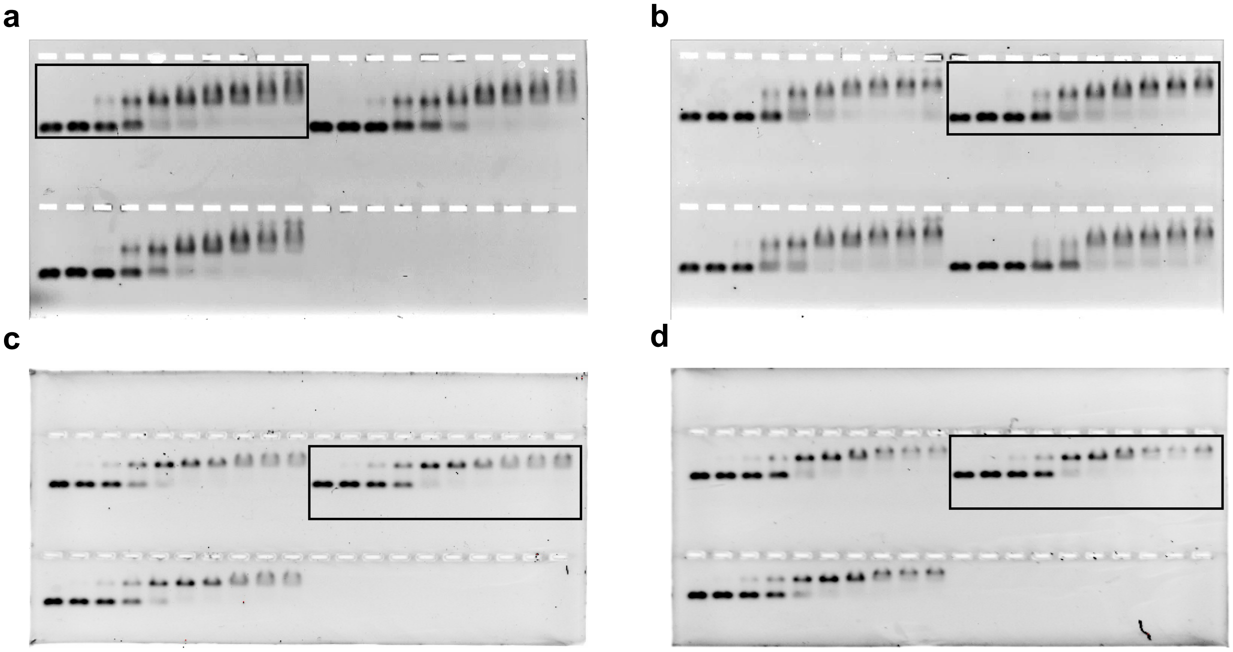

Figure S17. Uncropped gel images for Figure S4. (a) D1-5fC. (b) H1-5fC. (c) D2-5caC. (d) H2-5caC. Box regions indicate the cropped image shown in Figure S4.

Figure S18

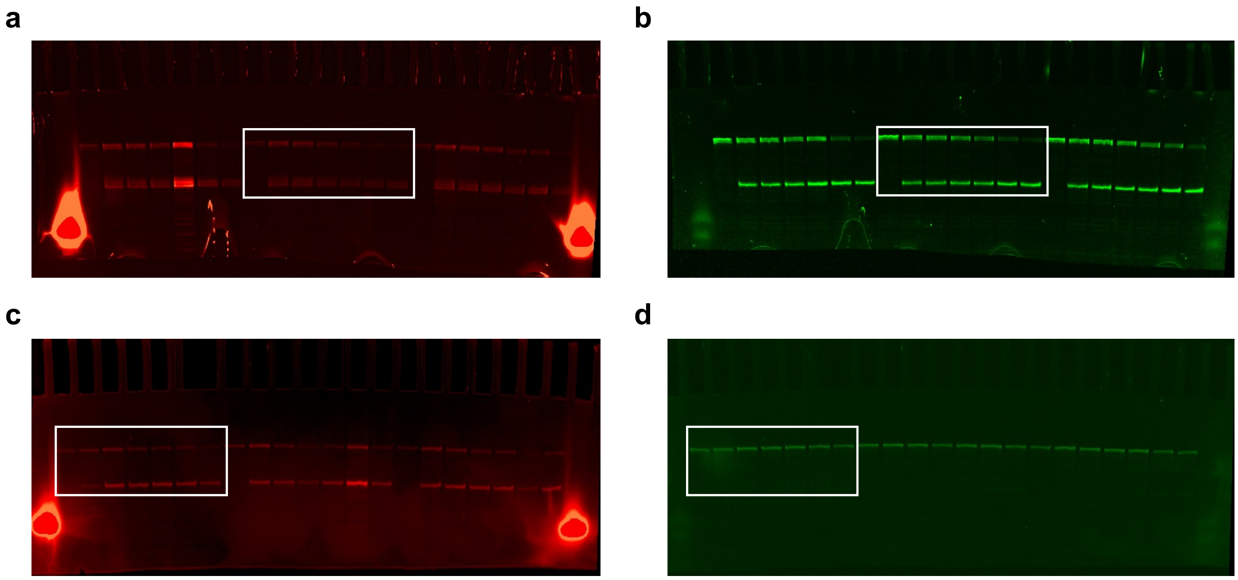

Figure S18. Uncropped gel images for Figure S7. (a) symGU (Cy5). (b) symGU (Cy3). (c) symGU-R (Cy5). (d) symGU-R (Cy3). Box regions indicate the cropped image shown in Figure S7.

Figure S19

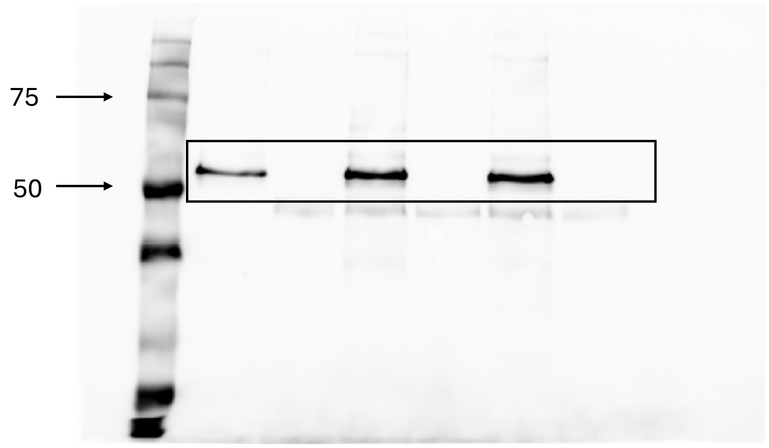

Figure S19. Uncropped gel image for Figure 7e. Frame indicates the cropped regions shown in Figure 7e. The calculated molecular weight of TDG is 46 kDa.

S2. Supplementary Tables

Table S1. Names and sequences of all oligonucleotides used in this work. U = deoxyuridine; T = deoxythymidine; U^F = 2′-deoxy-2′-fluoroarabinouridine; T^F = 2′-deoxy-2′-fluoroarabinothymidine; 5fC = 5-Formyl-2'-deoxycytidine, 5caC = 5-Carboxy-2'-deoxycytidine; /FAM/ = fluorescein /Cy5/ = sulfo-Cyanine 5 dye; /Cy3/ = sulfo-Cyanine 3 dye.

Substrate Name	Strand Name	Sequence Identity
D1-U	BZ-dUFWD	/FAM/TGAGGATGTATATATCTGA**U**GCGCCGGTGGAGC
	BZ-REV	GCTCCACCGGCGCGTACGATATATACATCCTCA
H1-U	BZ-dUFWD	/FAM/TGAGGATGTATATATCTGA**U**GCGCCGGTGGAGC
	BZ-rREV	GCUCCACCGGCGCGUACGAUAUAUACAUCCUCA
D1-T	BZ-dTFWD	/FAM/TGAGGATGTATATATCTGA**T**GCGCCGGTGGAGC
	BZ-REV	GCTCCACCGGCGCGTACGATATATACATCCTCA
H1-T	BZ-dTFWD	/FAM/TGAGGATGTATATATCTGA**T**GCGCCGGTGGAGC
	BZ-rREV	GCUCCACCGGCGCGUACGAUAUAUACAUCCUCA
D1-C	BZ-dCFWD	/FAM/TGAGGATGTATATATCTGA**C**GCGCCGGTGGAGC
	BZ-REV	GCTCCACCGGCGCGTACGATATATACATCCTCA
H1-C	BZ-dCFWD	/FAM/TGAGGATGTATATATCTGA**C**GCGCCGGTGGAGC
	BZ-rREV	GCUCCACCGGCGCGUACGAUAUAUACAUCCUCA
D1-5fC	BZ-5fCFWD	/FAM/TGAGGATGTATATATCTGA/**5fC**/GCGCCGGTGGAGC
	BZ-REV	GCTCCACCGGCGCGTACGATATATACATCCTCA
H1-5fC	BZ-5fCFWD	/FAM/TGAGGATGTATATATCTGA/**5fC**/GCGCCGGTGGAGC
	BZ-rREV	GCUCCACCGGCGCGUACGAUAUAUACAUCCUCA
D1-5caC	BZ-5caCFWD	/FAM/TGAGGATGTATATATCTGA/**5caC**/GCGCCGGTGGAGC
	BZ-REV	GCTCCACCGGCGCGTACGATATATACATCCTCA
H1-5caC	BZ-5caCFWD	/FAM/TGAGGATGTATATATCTGA/**5caC**/GCGCCGGTGGAGC
	BZ-rREV	GCUCCACCGGCGCGUACGAUAUAUACAUCCUCA

D1-U^F	BZ-dU^{F}FWD	/FAM/TGAGGATGTATATATCTGA/**U^F**/GCGCCGGTGGAGC
	BZ-REV	GCTCCACCGGCGCGTACGATATATACATCCTCA
H1-U^F	BZ-dU^{F}FWD	/FAM/TGAGGATGTATATATCTGA/**U^F**/GCGCCGGTGGAGC
	BZ-rREV	GCUCCACCGGCGCGUACGAUAUAUACAUCCUCA
D2-U	AD-dUFWD	/FAM/TGTGTCACCACTGCTCA**U**GTACAGAGCTG
	AD-REV	CAGCTCTGTACGTGAGCAGTGGTGACAC
H2-U	AD-dUFWD	/FAM/TGTGTCACCACTGCTCA**U**GTACAGAGCTG
	AD-rREV	CAGCUCUGUACGUGAGCAGUGGUGACAC
D2-T	AD-dTFWD	/FAM/TGTGTCACCACTGCTCA**T**GTACAGAGCTG
	AD-REV	CAGCTCTGTACGTGAGCAGTGGTGACAC
H2-T	AD-dTFWD	/FAM/TGTGTCACCACTGCTCA**T**GTACAGAGCTG
	AD-rREV	CAGCUCUGUACGUGAGCAGUGGUGACAC
D2-C	AD-dCFWD	/FAM/TGTGTCACCACTGCTCA**C**GTACAGAGCTG
	AD-REV	CAGCTCTGTACGTGAGCAGTGGTGACAC
H2-C	AD-dCFWD	/FAM/TGTGTCACCACTGCTCA**C**GTACAGAGCTG
	AD-rREV	CAGCUCUGUACGUGAGCAGUGGUGACAC
D2-U^F	AD-dU^{F}FWD	/FAM/TGTGTCACCACTGCTCA/**U^F**/GTACAGAGCTG
	AD-REV	CAGCTCTGTACGTGAGCAGTGGTGACAC
D2-T^F	AD-dT^{F}FWD	TGTGTCACCACTGCTCA/**T^F**/GTACAGAGCTG
	AD-REV	CAGCTCTGTACGTGAGCAGTGGTGACAC
TCF21-DNA	BZ-RL1	/Cy5/TCCTAGTGTCCACCAAATTCCTCAGCGCT**U**GCTCACCCTCCT CTACGGCCACGACTCTGGGAGTG
	BZ-RL2	/Cy3/CACTCCCAGAGTCGTGGCCGTAGAGGAGGGTGAGCGAGCGCT GAGGAATTTGGTGGACACTAGGA
TCF21-hybrid	BZ-RL1	/Cy5/TCCTAGTGTCCACCAAATTCCTCAGCGCT**U**GCTCACCCTCCT CTACGGCCACGACTCTGGGAGTG
	BZ-rRL(RNA)	GAGGGUGAGCGAGCG
	BZ-RL21	CTGAGGAATTTGGTGGACACTAGGA

	BZ-RL22	CACTCCCAGAGTCGTGGCCGTAGAG
TCF21-loop	BZ-RL1	/Cy5/TCCTAGTGTCCACCAAATTCCTCAGCGCT**U**GCTCACCCTCCTCTACGGCCACGACTCTGGGAGTG
	BZ-RL2con	CACTCCCAGAGTCGTGGCCGTAGAGTCTTAAAGTATGATACTGAGGAATTTGGTGGACACTAGGA
	BZ-rRL(RNA)	GAGGGUGAGCGAGCG
TCF21-flap	BZ-RL1	/Cy5/TCCTAGTGTCCACCAAATTCCTCAGCGCT**U**GCTCACCCTCCTCTACGGCCACGACTCTGGGAGTG
	BZ-RL21F	TCTTAAAGTATGATACTGAGGAATTTGGTGGACACTAGGA
	BZ-RL22	CACTCCCAGAGTCGTGGCCGTAGAG
	BZ-rRL(RNA)	GAGGGUGAGCGAGCG
TCF21-loopEX	BZ-RL1	/Cy5/TCCTAGTGTCCACCAAATTCCTCAGCGCT**U**GCTCACCCTCCTCTACGGCCACGACTCTGGGAGTG
	BZ-RL2EX	CACTCCCAGAGTCGTGGCCGTAGAGTCTTAAAGTATGATATCTTAAAGCACTATACTGAGGAATTTGGTGGACACTAGGA
	BZ-rRL(RNA)	GAGGGUGAGCGAGCG
symGU	BZ-NRL1	/Cy5/ATTGCCCTCGAGGTACCATGGATCCGATGT**U**GACCTCAAACCTAGACGAATTCCGTAGAC
	BZ-NRL2	/Cy3/GTCTACGGAATTCGTCTAGGTTTGAGGT**U**GACATCGGATCCATGGTACCTCGAGGGCAAT
symGU-R	BZ-NRL1	/Cy5/ATTGCCCTCGAGGTACCATGGATCCGATGT**U**GACCTCAAACCTAGACGAATTCCGTAGAC
	BZ-NRL2con	/Cy3/TAACGGGAGCTCCATGGTACCTTCTTGCATGTCCC**U**ATTTGGATCTGCTTAAGGCATCTG
	BZ-rNRL (RNA)	GAGGUCGACAUCGGA
